# The impact of dinotefuran application at different concentrations on soil microbial communities in vineyards

**DOI:** 10.3389/fmicb.2026.1784722

**Published:** 2026-03-09

**Authors:** Shaowei Cui, Feng Wang, Hui Wu, Luhang Li, Xiaoqing Huang, Decai Jin, Haijun Xiao, Weibin Li, Yongqiang Liu

**Affiliations:** 1 State Key Laboratory for Biology of Plant Diseases and Insect Pests, Institute of Plant Protection, Chinese Academy of Agricultural Sciences, Beijing, China; 2 School of Grassland Science, Beijing Forestry University, Beijing, China; 3 School of the Environment and Safety Engineering, Jiangsu University, Zhenjiang, China; 4 Research Center for Eco-Environmental Sciences, Chinese Academy of Sciences, Beijing, China

**Keywords:** application concentration, ecological assessment, grape phylloxera, insecticides, soil microbiomes

## Abstract

Soil microbiomes are essential for grapevine health and vineyard sustainability. Grape phylloxera poses a serious threat to the grape industry, although dinotefuran effectively controls this pest, the concentration-dependent effects of this insecticide on vineyard soil microbial communities remain unclear. Using high-throughput sequencing, this study examined the structural and functional responses of soil bacterial and fungal communities to varying concentrations of dinotefuran. Our results revealed that both 10 and 20% dinotefuran treatments significantly altered bacterial community structure without affecting bacterial alpha diversity. Fungal communities were more sensitive, showing significant structural changes, and significantly reduced alpha diversity (particularly richness) under the 20% treatment. In bacterial communities, high-concentration treatment reduced key soil health and nutrient cycling (e.g., *Kaistobacter*, *Solibacter*), and biocontrol (e.g., *Streptomyces*) group. The 10% treatment retained bacteria with potential ecological remediation (e.g., *Nocardioides*), whereas the relative abundance of potential stress-adapted bacteria (e.g., *Arthrobacter*) significantly increased. For fungi, high-concentration treatment reduced beneficial phosphate-solubilizing (e.g., *Mortierella*) and biocontrol (e.g., *Trichoderma*) fungi, while potential pathogenic fungal groups exhibited significantly higher relative abundances. Functionally, high-concentration insecticide treatment suppressed beneficial bacterial functions, including secondary metabolite synthesis, lipid metabolism, and microbial group behavior, while also reducing fundamental metabolic and genetic information processing activities. This treatment additionally increased the abundance of pathogenic and saprotrophic fungi, and decreased symbiotic fungi, with the relative abundances of plant pathogens showing a significant increasing under high doses. In contrast, low-concentration treatment enhanced bacterial detoxification pathways, whereas high-concentration treatment activated stress-response functions. These findings elucidate the dose-dependent responses of microorganisms to insecticides, and underscore the critical importance of rational pesticide application in maintaining soil ecological balance and vineyard sustainability.

## 1 Introduction

Soil microbial communities play a key role in almost all soil chemical and biological processes, and directly affect soil health, which underpins the sustainability of agricultural ecosystems and global food security (Bahram et al., 2018; Nadarajah and Rahman, 2023). In agroecosystems, soil microbes form complex associations with crop roots that can enhance nutrient uptake, increase resistance to biotic and abiotic stresses, and even influence crop quality and yield traits (Murali et al., 2021; Li et al., 2025). A stable microbial community is also critical for plant growth, physiology, and health (Zhang et al., 2024; Du et al., 2025). Importantly, the optimization of soil microbial communities is crucial for the sustainable production of agroecosystems (Cui et al., 2026). Additionally, as primary responders to soil contamination, soil microbial communities serve as sensitive indicators of exposure to pesticides, antibiotics, heavy metals, and other pollutants (Qin et al., 2023).

The community structure and function of soil microorganisms are typically influenced by various environmental factors, such as soil pH, moisture, temperature, organic matter content, and salinity (Tripathi et al., 2018; Garaycochea et al., 2020; Xu et al., 2021). Previous studies have shown that human activities, such as tillage, fertilization, irrigation, and pesticide application, exert strong selective pressures on soil microbiota, leading to changes in microbial composition, structure, and function (Yang et al., 2020; Walder et al., 2022; Wei et al., 2024). Such changes can affect soil health, nutrient availability, crop productivity, and ecosystem stability (Romero et al., 2025). Among these interventions, pesticide application has attracted increasing scientific attention for its effects on soil microbial diversity and functional potential.

Grape (*Vitis vinifera* L.), one of the world’s oldest cultivated fruits, is widely grown and consumed globally and has high economic value (Granato et al., 2016). According to the Food and Agriculture Organization (FAO, 2023), China’s harvested vineyard area reached 727,112 ha with an output of 16.2 million tonnes. The country consistently ranks among the world’s leading grape producers, and grape industry plays a pivotal role in China’s agricultural sector. However, in grape production, the damage caused by grape phylloxera (*Daktulosphaira vitifoliae* Fitch.) poses a serious threat to the healthy development of the industry (Powell, 2012; Arancibia et al., 2018). Grape phylloxera occurs in two distinct forms, root-galling and leaf-galling types, attack roots and leaves, respectively, with the root-galling form being the dominant group in China (Wang, 2017; Rubio et al., 2020). This pest is a root-feeding insect that causes root galls, impairs nutrient uptake, and predisposes grapevines to secondary infections by soil-borne pathogens (Wang, 2017). Moreover, grape phylloxera employs both asexual and sexual reproductive strategies, enabling its populations to expand rapidly in affected areas, leading to vine decline, vineyard degradation, and substantial economic losses (Lund et al., 2017; Yin et al., 2019). In recent years, the distribution and damage area of grape phylloxera in China have continued to expand. Chemical control is a highly effective measure for managing grape phylloxera. Active ingredients commonly used include neonicotinoids (e.g., dinotefuran and thiamethoxam) and organophosphates (Yin et al., 2019). However, the appropriate application dose of insecticides in vineyards, and their effects on vineyard soil ecology, particularly on soil microbial communities, have not been fully elucidated.

Insecticide application can pose notable environmental risks, including toxicity to aquatic organisms and pollinators (Sánchez-Bayo and Wyckhuys, 2019; Biondi et al., 2013), and disturbances to non-target soil microbial communities (Briceño et al., 2024). Previous studies have further demonstrated that insecticide use significantly alters soil microbial community structure and reduces its diversity (Liu et al., 2024). Thiamethoxam decreases bacterial diversity, reduces beneficial Actinobacteria, and increases Firmicutes linked to pollutant degradation (Wu et al., 2021). Pyrethroids promote copiotrophic bacteria and actinomycetes while inhibiting fungal growth (Borowik et al., 2023). Insecticides also disrupt microbial functions, impairing ecosystem services such as soil carbon cycling and organic matter stability (Sim et al., 2022), with organochlorine pesticides showing ecotoxic effects on native microbiomes (Egbe et al., 2021). The concentration of application critically modulates these impacts, higher doses of cypermethrin cause stronger and more persistent suppression of enzyme activity and microbial diversity, while low doses allow partial recovery over time (Tejada et al., 2015; Wu et al., 2018). These studies elucidate the impacts of insecticides on non-target soil microorganisms, and highlight the influence of insecticide dosage in this context.

Dinotefuran is a third-generation neonicotinoid systemic insecticide (Matsuda et al., 2020). It exhibits high acute toxicity to non-target pollinators such as bees, and can contaminate aquatic systems through leaching and runoff (Sánchez-Bayo and Wyckhuys, 2019). Although mammalian acute toxicity is relatively low, its environmental persistence and metabolites raise concerns over chronic exposure, potentially affecting ecosystem functions and human health (Goulson, 2013; Chagnon et al., 2015). In soil, dinotefuran application significantly reduces bacterial diversity and richness (Yamaguchi et al., 2021), enhances antagonistic interactions among soil organisms, and increases soil organic carbon mineralization (Yu et al., 2021). However, the impact of dinotefuran application on soil microbial communities in vineyard ecosystems remains poorly understood.

Soil microorganisms play a critical role in grapevine health and vineyard sustainability. Although dinotefuran shows effective control against grape phylloxera, its concentration-dependent effects on non-target soil microbial communities in vineyards remain unclear. This study used high-throughput sequencing to investigate the effects of dinotefuran application at different concentrations on the composition and function of bacterial and fungal communities in vineyard soils. The findings will provide important insights for rational insecticide use, helping to mitigate negative impacts on vineyard sustainability.

## 2 Materials and methods

### 2.1 Experimental design and sampling

This study was conducted in a vineyard (110°52′E, 25°45′N), located in Guilin City, Guangxi Zhuang Autonomous Region. The experimental site located at 548 m elevation, has a humid subtropical monsoon climate characterized by an average annual temperature of 18.2 °C, annual precipitation of 1942.9 mm, 1297.0 h of sunshine, and 79% relative humidity. The soil is red soil, the vineyard was planted with “Kyoho” grapevines, and follows conventional cultivation practices.

The experiment consisted of two dinotefuran (99.1% purity, Sino-Agri Union Co., Ltd., China) concentration treatments: 10% dinotefuran (T10) and 20% dinotefuran (T20). Both treatments were applied according to the manufacturer’s instructions, using water as the control (T0). Apart from the experimental treatments, all vineyard plots received identical management practices. Each treatment was replicated six times. Each treatment was replicated across six grapevines.

Soil samples were collected from root zone of each grapevine 15 days after application, taken at a depth of 5–15 cm. Plant debris and gravel were removed, and the samples were passed through a 2 mm sieve. The soil samples were placed in sterile self-sealing bags, and transported to the laboratory within 12 h using a cooler. The samples were stored at −80 °C until further analysis.

### 2.2 DNA extraction, PCR and sequencing

Soil samples were collected in small self-sealing bags, fitted with ventilation tubes, and freeze-dried using a freeze dryer (FD-1C-50, Beijing Boyikang Experimental Instrument Co., Ltd.). Subsequently, 0.5 g of each soil sample was weighed, and total DNA was extracted using the FastDNA^™^ Spin Kit for Soil (MP Biomedicals, United States) following the manufacturer’s instructions. The DNA concentration of each sample was measured with a NanoDrop ND-1000 spectrophotometer (Thermo Fisher Scientific, United States), and the DNA was stored at −40 °C.

PCR amplification and high-throughput sequencing were performed following the methods described by Cui et al. (2026). The16S rRNA gene was amplified using primers 515F (5′-GTGCCAGCMGCCGCGG-3′)/806R (5′-GGACTACHVGGGTWTCTAAT-3′), and the ITS region was amplified with primers gITS7F (5′-GTGARTCATCGARTCTTTG-3′)/ITS4 (5′-TCCTCCGCTTATTGATATGC-3′). Each primer pair contained a unique 12-bp barcode. The reaction mixture consisted of 5 μL 10 × PCR buffer, 4 μL dNTP, 0.5 μL DNA polymerase (TaKaRa Biotech, Beijing, China), 1.5 μL 10 μM each primer, and 1 μL DNA template. The thermal cycler (Bio-Rad Laboratories, CA, United States) was initial denaturation at 94 °C for 1 min; 30 cycles of denaturation at 94 °C for 20 s, annealing at 57 °C for 25 s, and extension at 72 °C for 45 s for 16S rRNA or 33 cycles for ITS. The PCR products were separated by electrophoresis in a 1% agarose gel, and purified with the E.Z.N.A. Gel Extraction Kit (Omega Bio-tek, Norcross, GA, United States) following the manufacturer’s instructions. After library construction for all samples, they were pooled in equimolar amounts, and sequenced by Magigene Biotechnology Co., Ltd. (Guangzhou, China).

### 2.3 Microbiome analyses and data statistical analysis

Sequencing raw data were processed using the Galaxy online analysis platform^1^ (Feng et al., 2024). Paired-end reads were merged using FLASH, and low-quality sequences were filtered out. Chimeric sequences were removed, and the remaining sequences were clustered into operational taxonomic units (OTUs) at a 97% similarity threshold using UPARSE. Taxonomic assignment was performed using the SILVA SSU r138.1 database for bacteria, and the UNITE 8.3 database for fungi.

Alpha diversity was evaluated using the Shannon, observed richness, and Pielou evenness. Constrained principal coordinate analysis (CPCoA) based on Bray–Curtis distance was conducted (Zgadzaj et al., 2016), and PERMANOVA was used to test for differences among groups. Community composition was analyzed at the phylum and genus levels. Significant biomarkers were identified using linear discriminant analysis effect size (LEfSe) on Galaxy online analysis platform. Functional predictions for both bacterial and fungal communities were conducted using Tax4Fun2 and FunGuild, respectively (Nguyen et al., 2016; Wemheuer et al., 2020). Both analyses were based on the unified OTU table and corresponding taxonomic annotation table generated from all treatment samples. The specific methods for microbial data analysis followed previous studies (Cui et al., 2024).

All statistical analysis were performed using SPSS Statistics 27 (IBM, Armonk, NY, United States). One-way ANOVA was employed to test for significant differences among treatments in alpha diversity indices, relative abundances at the phylum and genus levels, and functional groups. Tukey’s HSD test (*p* < 0.05) was used for multiple comparisons.

## 3 Results

### 3.1 Diversity of soil microbial communities

**Figure 1:**
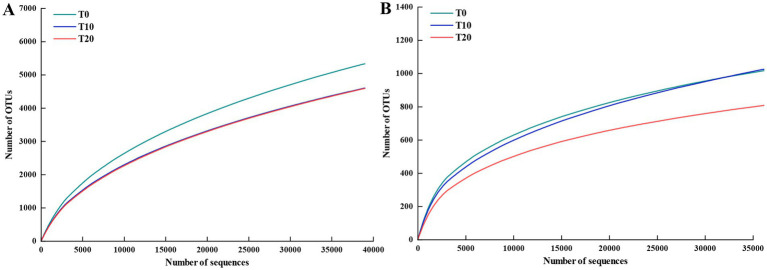
Rarefaction curves of soil bacterial **(A)** and fungal **(B)** communities.

A total of high-quality 2,288,085 bacterial sequences and 3,903,025 fungal sequences were retained across soil samples. Following resampling for data normalization, 23,426 bacterial reads and 4,325 fungal reads were retained. Bacterial and fungal rarefaction curves reached asymptotes (Figure 1), indicating adequate sequencing depth.

**Figure 2:**
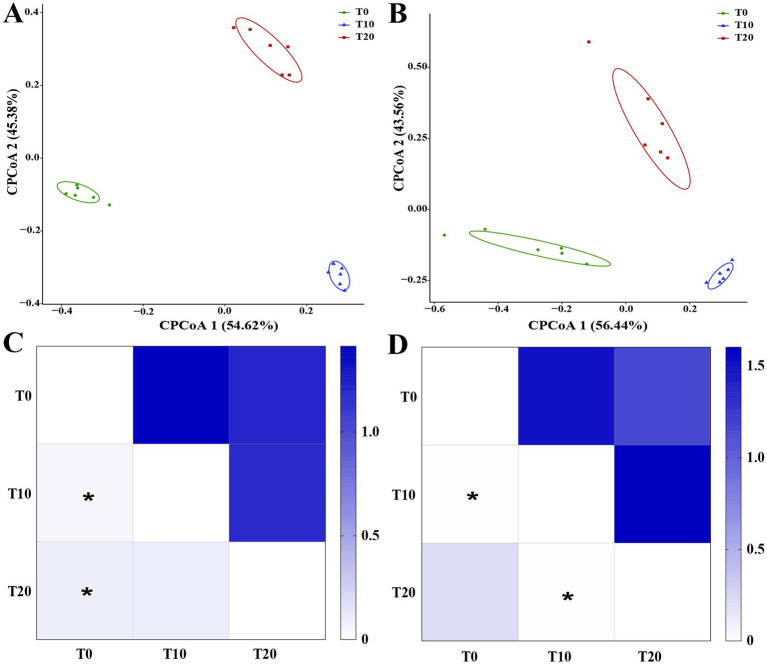
Constrained principal coordinate analysis (CPCoA) of soil bacterial **(A)** and fungal **(B)** communities under different dinotefuran concentrations. **(C,D)** Present PERMANOVA heatmaps for bacterial and fungal communities, respectively. The upper triangle displays *F* values, the lower triangle shows *p*-values in heatmaps, and * indicate *p* < 0.05.

Constrained principal coordinate analysis (CPCoA) was used to assess the effects of different insecticides on soil microbial communities. The results indicated that insecticide application significantly altered the structure of both soil bacterial and fungal communities. As shown in Figure 2, for bacteria, CPCoA1 explained 54.62% of the variance and CPCoA2 explained 45.38% (Figure 2A), while for fungi, CPCoA1 explained 56.44% and CPCoA2 explained 43.56% (Figure 2B). Samples of both bacteria and fungi were clearly separated into three groups. PERMANOVA analysis revealed that soil bacterial communities in the T10 and T20 treatments were significantly different from those in T0 (Figure 2C; *p* < 0.05). For fungal communities, significant differences were observed between T20 and T0, as well as between T20 and T10 (Figure 2D; *p* < 0.05).

**Figure 3:**
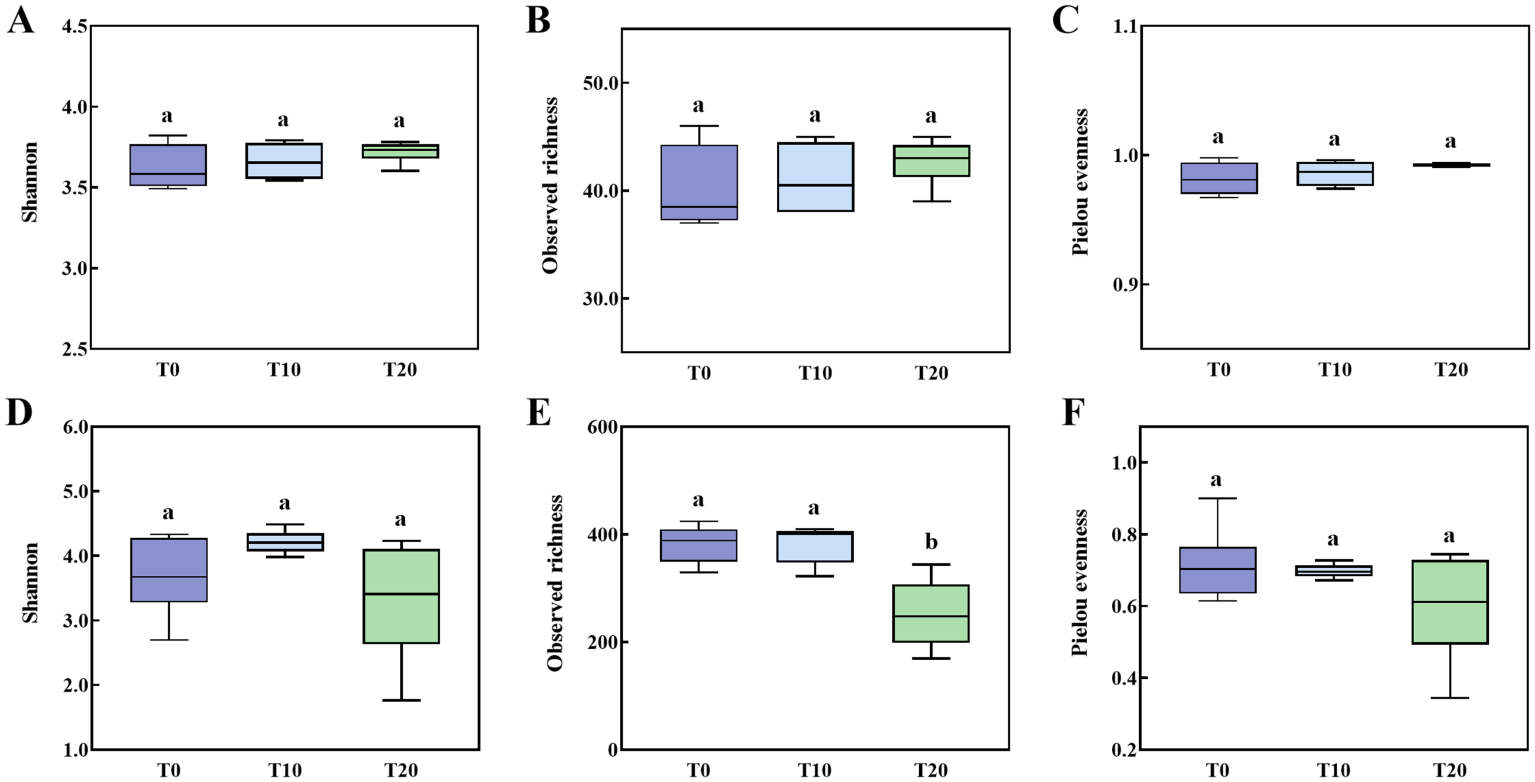
Alpha diversity of soil bacterial **(A–C)** and fungal **(D–F)** communities under different dinotefuran concentrations treatments.

The application of dinotefuran had a minimal impact on the alpha diversity of bacterial communities in grapevine soil. No significant differences were observed in Shannon index, richness index, or evenness index of bacterial communities across different concentration treatments (Figures 3A–C; *p* > 0.05). In contrast, dinotefuran application significantly affected the alpha diversity of fungal communities. Compared with the water control (T0) and the 10% dinotefuran treatment (T10), the 20% insecticide treatment (T20) resulted in reduced Shannon index, richness index, and evenness index of the soil fungal community. Specifically, the richness index was significantly lower in T20 than in T0 and T10 (Figures 3D–F; *p* < 0.05).

### 3.2 Microbial composition

**Figure 4:**
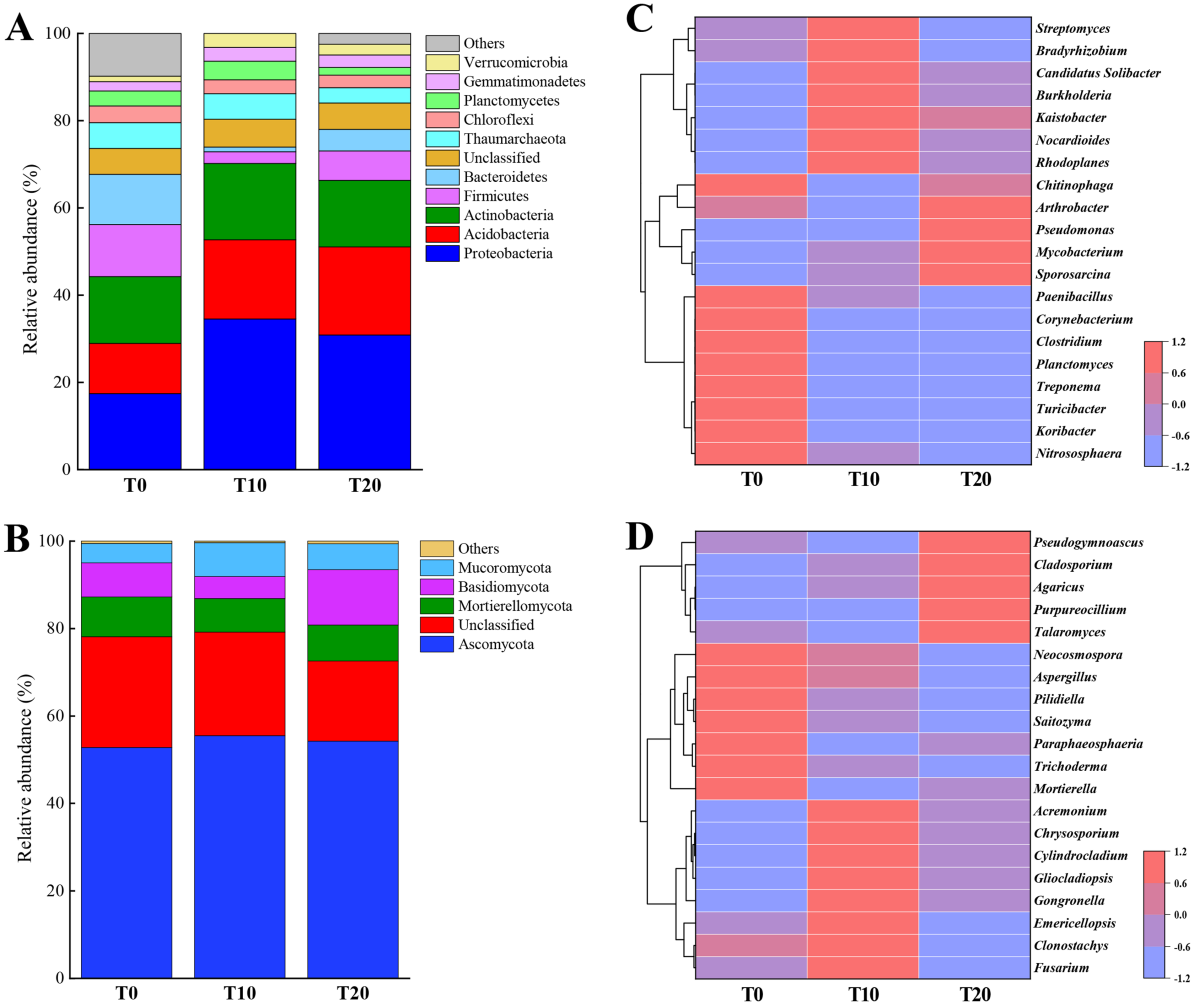
Relative abundances of soil bacteria and fungi at phylum and genus levels under different treatments. **(A,B)** Show the relative abundances of bacterial and fungal communities at the phylum level, respectively. **(C,D)** Present heatmaps of the top 20 genera of bacteria and fungi, respectively. “Others” represents the sum of the relative abundances of community members with less than 2% abundance across all soil samples.

The soil bacterial community comprised 28 phyla, with the dominant phyla being Proteobacteria, Acidobacteria, Actinobacteria, Firmicutes, Bacteroidetes, Chloroflexi, and Planctomycetes (Figure 4A). Firmicutes and Bacteroidetes exhibited significantly higher relative abundances in the T0 treatment, while the relative abundance of Planctomycetes was significantly lower in T20 (*p* < 0.05). At the fungal phylum level, the dominant groups were Ascomycota, Mortierellomycota, Basidiomycota, and Mucoromycota (Figure 4B). Basidiomycota showed a significantly higher relative abundance in T20 (*p* < 0.05).

A total of 107 bacterial genera were identified. Figure 4C displays the top 20 bacterial genera ranked by relative abundance. Compared with T10 and T20, *Koribacter*, *Turicibacter*, *Treponema*, *Planctomyces*, *Clostridium*, and *Corynebacterium* had a significantly higher relative abundance in control (*p* < 0.05). The relative abundances of *Nitrososphaera* and *Paenibacillus* were significantly higher in T0 than in T20 (*p* < 0.05). *Chitinophaga* had a significantly higher relative abundance in T0 than in T10. Several beneficial bacterial genera maintained significantly higher relative abundances in the T10, such as *Kaistobacter*, *Solibacter*, and *Streptomyces*. Additionally, some genera with ecological remediation functions were present in insecticide-treated soils, *Burkholderia* and *Nocardioides* showed significantly higher relative abundances in T10, whereas the relative abundances of *Arthrobacter* and *Mycobacterium* were significantly higher in T20.

A total of 484 fungal genera were identified, with the top 20 genera shown in Figure 4D. *Mortierella* and *Trichoderma* had significantly higher relative abundances in T0 (*p* < 0.05). *Fusarium* and *Gongronella* had significantly higher relative abundances in T10 (*p* < 0.05). *Cladosporium* and *Talaromyces* showed significantly hgigher relative abundances in T20 (*p* < 0.05). Some beneficial fungal genera maintained relatively high abundances in T10, such as *Clonostachys* and *Gliocladiopsis*. Meanwhile, certain pathogenic fungi, such as *Cylindrocladium*, had significantly lower relative abundances in T0.

**Figure 5:**
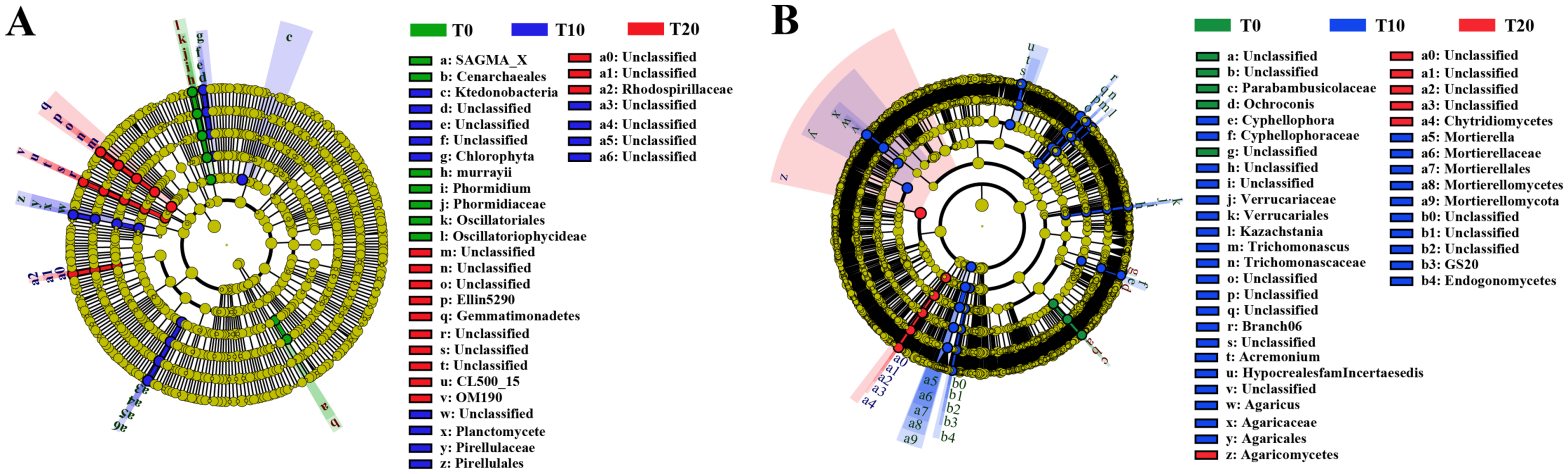
LEfSe analysis of soil bacterial **(A)** and fungal **(B)** communities under different treatments.

LEfSe analysis was used to identify taxa that showed significant differences in bacterial and fungal communities among the different treatments (LDA score >3, *p* < 0.05; Figure 5). For bacterial communities, the LEfSe results indicated that 7, 13, and 13 bacterial biomarkers were identified in T0, T10, and T20, respectively (Figure 5A). The relative abundances of *Phormidium* and Cenarchaeales were significantly higher in T0-treated soil, Pirellulaceae showed a significantly higher relative abundance in T10, and Rhodospirillaceae was significantly higher in T20.

For fungal communities, 5, 30, and 6 fungal biomarkers were identified in T0, T10, and T20, respectively (Figure 5B). Notably, *Ochroconis* exhibited a significantly higher relative abundance in T0. In T10-treated soil, *Trichomonascus*, *Cyphellophora*, *Acremonium*, Hypocreales fam incertae sedis, *Mortierella*, *Kazachstania*, and *Agaricus* had significantly higher relative abundances. In contrast, Chytridiomycetes and Agaricomycetes showed significantly higher relative abundances in T20.

### 3.3 Functional profiling of microbial composition

**Figure 6:**
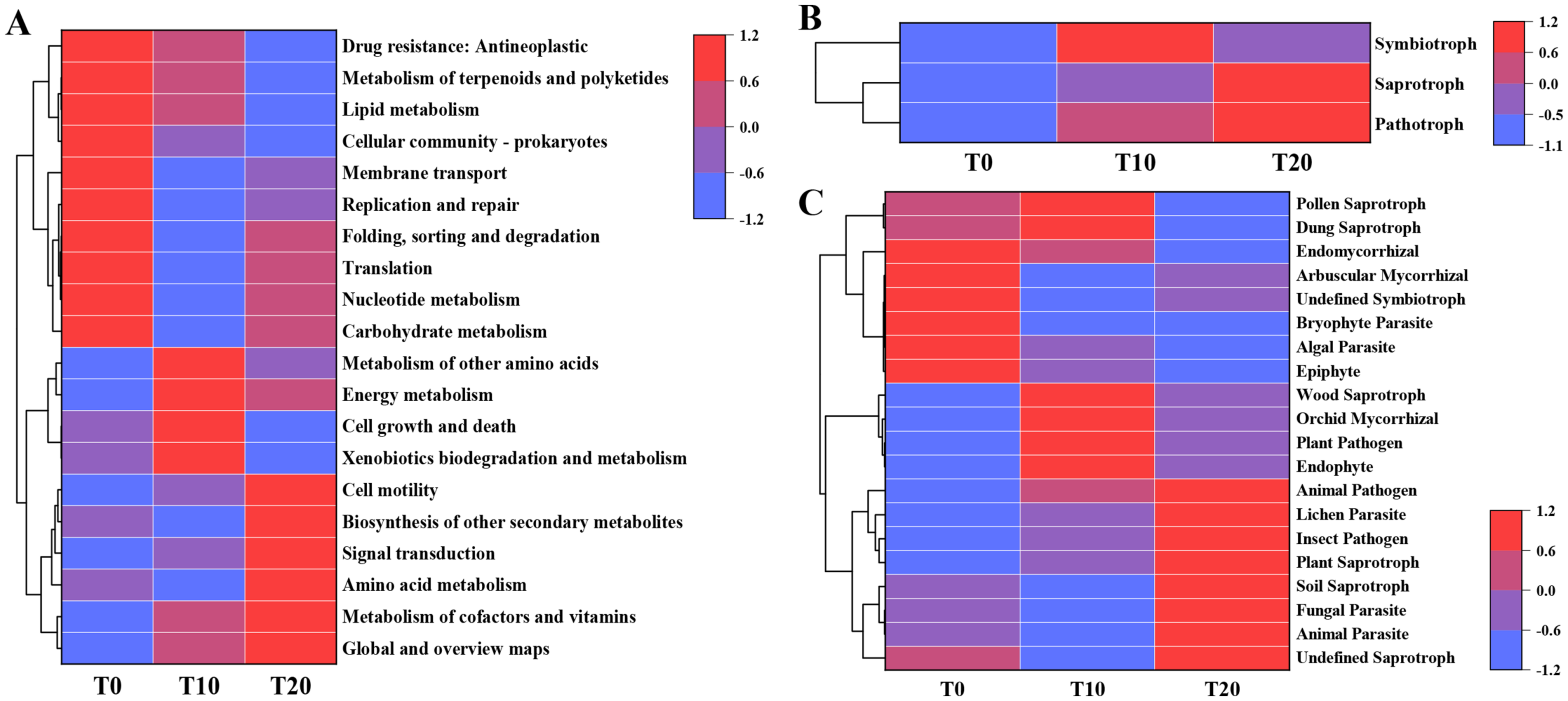
Heatmap of the functional profiling for soil bacterial **(A)** and fungal **(B,C)** communities.

The potential functional profiles of bacterial communities were predicted using Tax4Fun2 analysis (Figure 6A). A total of 337 KEGG pathways were identified, encompassing six major functional categories: metabolism, environmental information processing, cellular processes, human diseases, genetic information processing, and organismal systems. Among these, metabolism exhibited the highest relative abundance across all samples (74.61–75.37%), followed by environmental information processing (10.13–10.75%). The relative abundances of both environmental information processing and genetic information processing were significantly higher in T0 (*p* < 0.05). Regarding bacterial functional groups, the relative abundances of metabolism of terpenoids and polyketides, lipid metabolism, and cellular community–prokaryotes were significantly higher in the T0 group compared to the T20 group (*p* < 0.05). Functional groups such as membrane transport, replication and repair, folding sorting and degradation, translation, nucleotide metabolism, and carbohydrate metabolism showed significantly higher relative abundances in T0 than in T10 (*p* < 0.05). Additionally, cell growth and death and xenobiotics biodegradation and metabolism were significantly more abundant in T10 than in T20 (*p* < 0.05). Notably, cell motility and signal transduction were significantly enriched in T20 compared to T0, while biosynthesis of other secondary metabolites and amino acid metabolism were higher in T20 than in T10.

The FUNGuild tool was employed to predict fungal functional groups. The trophic mode prediction coverage was 83.01%, and the functional guild annotation coverage reached 78.18%, indicating that most microorganisms could be matched to known functional information in the current databases, and the analysis results show strong representativeness for the entire community. Classifying taxa into three trophic modes: pathotroph, saprotroph, and symbiotroph (Figure 6B). The relative abundances of pathotrophs and saprotrophs were significantly higher in the T20 group compared to the T0 group (*p* < 0.05). Specifically (Figure 6C), endomycorrhizal fungi, bryophyte parasites, animal pathogens, and epiphytes showed significantly higher relative abundances in T0 than in T20 (*p* < 0.05), while arbuscular mycorrhizal fungi and undefined symbiotrophs had significantly higher relative abundances in T0 than in T10 (*p* < 0.05). Dung saprotrophs, wood saprotrophs, and endophytes were significantly enriched in T10 (*p* < 0.05). In contrast, plant saprotrophs, undefined saprotrophs, soil saprotrophs, and insect pathogens were significantly showed higher relative abundances in the T20 (*p* < 0.05). Notably, the relative abundance of plant pathogens was higher in both T10 and T20, with T20 showing a significant increase compared to T0 (*p* < 0.05).

## 4 Discussion

The soil microbial community observed in the control treatment of this study exhibited a composition consistent with typical vineyard ecosystems globally. At the bacterial phylum level, the community was dominated by Proteobacteria, Actinobacteria, Acidobacteria, Bacteroidetes, Firmicutes, and Chloroflexi, a profile that aligns with reports from vineyards in France, Italy, Portugal, Switzerland, Israel, and China (Longa et al., 2017; Unc et al., 2021; Quiquerez et al., 2022; Wei et al., 2023; Steiner et al., 2024; Teixeira et al., 2024). Notably, Proteobacteria, Actinobacteria, and Acidobacteria were consistently among the most abundant phyla across these regions. Similarly, the fungal community was characterized by the predominance of Ascomycota, Mortierellomycota, and Basidiomycota, which are also frequently identified as dominant fungal phyla in vineyards from Italy, Portugal, Switzerland, and China (Coller et al., 2019; Zhang et al., 2022; Teixeira et al., 2025; Cui et al., 2026). This broad geographic consistency in microbial composition confirms that the baseline community in our experimental soil is representative of vineyard systems, thereby supporting the ecological relevance and generalizability of the pesticide-induced shifts reported in this study.

### 4.1 Insecticide application alters microbial community structure, with fungi exhibiting greater sensitivity than bacteria

Soil microbial communities in vineyards play a crucial role in grape growth and health (Wei et al., 2024; Cui et al., 2026). However, the composition of microbial communities is often influenced by multiple factors. The application of pesticides exerts strong selective pressure on soil microbial communities, subsequently altering their composition and functions (Walder et al., 2022), thereby affecting the sustainable production of vineyards. Our findings indicated that insecticides application significantly altered both bacterial and fungal communities in vineyard soils. Notably, fungal communities exhibited more pronounced changes in structure and diversity compared to bacterial communities, particularly under high-concentration dinotefuran treatment, which led to a marked reduction in fungal richness. The stronger response of fungi to insecticide disturbance aligns with previous studies highlighting the heightened sensitivity of eukaryotic microorganisms to agrochemicals. Compared to bacteria, fungi generally have slower growth rates and more complex reproductive strategies, making their communities more challenging to recover after disturbance (Romdhane et al., 2019). Furthermore, many soil fungi are hyphal-forming organisms, and their extensive surface contact with soil particles and water films increases their exposure to pesticides (Jacobsen and Hjelmso, 2014).

However, compared to fungi, bacterial communities showed no significant changes in alpha diversity indices, reflecting their higher functional redundancy and adaptive capacity. Bacteria are better equipped to cope with external environmental stress through mechanisms such as rapid generational turnover, horizontal gene transfer, and metabolic plasticity (Cycon et al., 2019). Additionally, bacterial communities often consist of numerous species with similar functions, meaning the loss of a single species can be compensated by other groups, thereby maintaining overall functional stability (Bindraban, 2025). The observation in this study that bacterial community structure changed while diversity remained unchanged suggests that bacteria may adapt to pesticide stress through interspecies substitution or adjustments in metabolic pathways. However, such structural adjustments could still impact the efficiency of specific ecological processes, such as nitrification and nitrogen fixation.

The effects of different concentrations of dinotefuran on microbial communities showed a clear concentration-gradient response, with high-concentration treatments exhibiting the most pronounced inhibition on fungal communities. This dose–response relationship provides a critical basis for determining appropriate application concentrations. Long-term or high-dose use of neonicotinoid pesticides may drive soil microbial communities toward tolerant yet functionally simplified states, thereby compromising soil health and ecosystem services (Huang et al., 2018). In particular, the decline in fungal diversity may reduce the soil’s resistance to disturbance and resilience, increasing the risk of soil-borne disease outbreaks.

### 4.2 Pesticide application alters microbial composition, high concentrations reduce beneficial microorganisms and increase disease risk

This study revealed that high-concentration dinotefuran treatment not only altered microbial community structure but also suppressed beneficial microorganisms while promoting the proliferation of potential pathogens. At the phylum level, the high-concentration treatment significantly reduced the relative abundance of Planctomycetes, a key bacterial phylum involved in nitrogen cycling that plays a vital role in soil nitrogen transformation, particularly in anaerobic ammonium oxidation (Wiegand et al., 2018). This decline may impair soil nitrogen use efficiency. Concurrently, the abundances of beneficial bacterial genera essential for nutrient cycling and soil health maintenance, such as *Kaistobacter*, *Solibacter*, and *Streptomyces*—were significantly decreased under high-concentration treatment. *Streptomyces* is involved not only in organic matter decomposition but also produces various antibiotic substances that suppress pathogen growth (Cycon et al., 2019). Its reduction can directly weaken the soil’s biocontrol capacity.

Fungal communities exhibited greater sensitivity to pesticide application than bacterial communities. High-concentration treatment led to a significant decrease in *Mortierella*, which solubilizes phosphate, and *Trichoderma*, a biocontrol agent (Sang et al., 2022; Chen et al., 2025). *Trichoderma* is an important biocontrol fungus that suppresses pathogens through multiple mechanisms, including nutrient competition, mycoparasitism, and induction of plant resistance (Chen et al., 2025). Its decline may undermine the soil’s natural disease-suppressive ability. Meanwhile, high-concentration treatment had significantly higher relative abundances of *Cladosporium*, *Talaromyces*, and *Fusarium*. These taxa may also include endophytic microorganisms that could be beneficial. According to existing research, *Cladosporium* includes more species that can cause leaf spot diseases (Huang et al., 2025), and *Fusarium* encompasses several important pathogens responsible for grapevine wilt and root rot (Cruz et al., 2025). The increased relative abundance of these taxa may elevate the potential disease risk in vineyards, though this inference warrants further validation through subsequent experiments involving microbial isolation and functional characterization. Furthermore, LEfSe analysis confirmed differential microbial responses across concentration treatments. In bacterial communities, *Phormidium* (involved in primary production) and *Cenarchaeales* (involved in ammonia oxidation) had significantly higher relative abundances in the control treatment, while the relative abundances of *Rhodospirillaceae* was significantly higher under high-concentration treatment, is primarily associated with stress adaptation, reflecting a shift toward tolerance-related functional traits.

Notably, genera with bioremediation capabilities, such as *Burkholderia* and *Nocardioides*, had significantly higher relative abundances under moderate-concentration treatment, whereas community dominance shifted toward stress-tolerant genera like *Arthrobacter* and *Mycobacterium* under high-concentration treatment. This functional group transition suggests that high pesticide pressure selects for highly adaptive microbial taxa, which may also represent a soil response to recruit microorganisms for ecosystem recovery (Romdhane et al., 2019).

### 4.3 Enhanced environmental remediation functions of soil microorganisms following pesticide application

The high-concentration dinotefuran treatment significantly inhibited beneficial metabolic functions of soil bacteria, including the metabolism of terpenoids and polyketides (involved in the synthesis and degradation of secondary metabolites), lipid metabolism, and the cellular community function of prokaryotes, which reflects microbial group behavior. These functions are typically closely associated with soil organic matter transformation, microbial interactions, and ecosystem stability (Bindraban, 2025). The decline in their abundance suggests that high pesticide concentrations may disrupt bacterial anabolic processes and quorum sensing, thereby weakening the biochemical functional diversity of the soil.

Concurrently, fundamental metabolic and genetic information processing functions, such as membrane transport, carbohydrate metabolism, nucleotide metabolism, and replication and repair, are generally downregulated following pesticide application. This indicates that pesticide stress may reduce the energy allocated by microbial cells for maintaining basic life activities or induce pressure related to DNA damage repair (Cycon et al., 2019). Notably, the high-concentration treatment significantly increased the abundance of stress-responsive functions such as cell motility and signal transduction, which may represent survival strategy adjustments by microorganisms to evade stressful environments or initiate adaptive regulation (Romdhane et al., 2019). Furthermore, in the moderate-concentration treatment, the function of xenobiotic biodegradation and metabolism was significantly enhanced. This suggests that this concentration may activate the pesticide degradation potential of certain microorganisms, reflecting the metabolic resilience of microbial communities to low-dose stress (Huang et al., 2018).

Functional prediction coverage is a core metric for evaluating the reliability of functional inferences derived from amplicon sequencing, and its level directly impacts the robustness of research conclusions and the boundaries of ecological interpretation (Nguyen et al., 2016). In this study, the coverage rates for fungal trophic mode prediction and functional guild annotation were 83.01 and 78.18%, respectively, indicating that the functions of most taxa are represented in existing databases, thereby enhancing the credibility of the prediction results. However, the approximately 17–22% of unannotated OTU suggests that the community may contain unknown or novel functional groups, or that functional redundancy may exist between annotated and unannotated taxa, potentially leading to an underestimation of the actual functional disturbances (Lladó et al., 2016). High-concentration dinotefuran significantly altered the trophic functional structure of fungal communities, manifesting as an increase in the relative abundances of pathotrophs and saprotrophs and a decrease in symbiotrophs. Symbiotic fungi, particularly arbuscular mycorrhizal fungi, enhance plant nutrient uptake and stress tolerance, their reduction can directly impact the health and growth of grapevines (Streletskii et al., 2022).

Interestingly, the abundance of potential plant pathogens showed an increasing trend in both 10% dinotefuran and 20% dinotefuran, with the latter being significantly higher than the control. This clearly indicates that pesticide application may indirectly elevate the risk of plant disease occurrence by altering fungal community composition (Gqozo et al., 2020). Additionally, the relative abundances of plant saprotrophs and soil saprotrophs were significantly higher in 20% dinotefuran treatment suggests potential changes in soil organic matter decomposition pathways, which could further influence nutrient cycling processes. It is noteworthy that dung saprotrophs, wood saprotrophs, and endophytes had significantly higher relative abundances in 10% dinotefuran treatment indicates that fungal communities under moderate-concentration treatment retain certain organic matter decomposition and plant-beneficial functions. However, this is accompanied by a potential increase in pathogenic taxa, exhibiting a “functional trade-off” characteristic.

Integrating the above findings, during the application of insecticides to control grape phylloxera, it is essential to use pesticides rationally and avoid excessive application that could disrupt soil microbial communities and increase potential disease risks in vineyards. Even moderate pesticide application warrants attention to its long-term latent effects on soil microbial functions. Future research could employ metagenomic technologies to further validate the actual expression levels of these predicted functions, confirm the functional roles of potentially beneficial and pathogenic microorganisms through microbial isolation, and elucidate the response mechanisms of key metabolic pathways under pesticide stress.

## 5 Conclusion

In conclusion, this study reveals that insecticide application at varying concentrations significantly alters the structure and function of vineyard soil microbial communities, with fungi demonstrating higher sensitivity than bacteria. While high-concentration dinotefuran did not affect bacterial diversity, it markedly reduced fungal alpha-diversity. The treatments induced substantial compositional shifts, notably decreasing beneficial microbial taxa and increasing potential pathogenic fungi, especially under high concentrations, thereby elevating potential disease risk despite the retention of some beneficial fungal functions at lower doses. Functionally, insecticides broadly suppressed beneficial bacterial metabolism while enhancing fungal pathogenicity and saprotrophic activity, though pollutant-degrading and bio-remediating bacterial taxa were enriched. These findings elucidate the dose-dependent microbial responses to insecticides and emphasize the critical need for optimized pesticide management to maintain soil ecological balance and vineyard sustainability.

## Data Availability

The datasets presented in this study can be found in online repositories. The names of the repository/repositories and accession number(s) can be found at: https://www.ncbi.nlm.nih.gov/, PRJNA1400748.

## Footnotes

1
https://dmap.denglab.org.cn
